# The Challenge of Sustaining Long-term Care in Aging Societies: Lessons From Japan and Spain Comment on "Financing Long-term Care: Lessons From Japan"

**DOI:** 10.15171/ijhpm.2019.143

**Published:** 2020-01-01

**Authors:** Raúl Del Pozo-Rubio, Dolores Jiménez-Rubio

**Affiliations:** ^1^Faculty of Social Sciences, University of Castilla-La Mancha, Cuenca, Spain.; ^2^Department of Applied Economics, Faculty of Economics and Business Sciences, University of Granada, Granada, Spain.

**Keywords:** Long-term Care, Cash Benefits, Informal Care, Japan, Spain

## Abstract

This article compares the provision of long-term care (LTC) in Japan and Spain, two countries with similar demographic structures but which address the provision of LTCs in very different ways. Both countries provide universal LTC. However, Japan has developed a generous benefit package of formal services for dependents to alleviate the care burden on the family, but provides no cash benefits. In Spain, on the other hand, cash allowances are the norm rather than the exception in the practical implementation of LTC services. After discussing the necessary delineation of LTC in response to future sociodemographic challenges, we discuss LTC system characteristics and the recent cost containment reforms implemented in Japan and Spain. Finally, we consider the lessons that may be drawn from each country’s experience and the reforms that must be undertaken in order ensure the sustainability of LTC provision in other countries with incipient or more developed LTC systems. In addition, since Japan and Spain are both faced with challenging demographic projections, it is important for each country to learn from the other’s initiatives and reforms.

## Defining and Delineating Long-term Care

In his Editorial, Ikegami presents some important reflections on systems of long-term care (LTC) and the ways in which they are addressed, and provides an excellent summary of the history of LTC provisions in Japan.^1^ It is striking that Japan and Spain, where life expectancy is among the highest in the world,^2^ and which have very similar demographic structures, have addressed the provision of LTC in such different ways despite relying in both cases on geographically devolved systems of LTC (to regions in the case of Spain and municipalities in the case of Japan). The aim of the present article is to comment on the short but intense evolution of LTC in Spain and to compare it with the situation in Japan, as described by Ikegami. We conclude by discussing the lessons that can be drawn from each country’s experience and outline the reforms that may be necessary in response to future sociodemographic challenges.

Ikegami begins by discussing the foundations of LTC systems, defining the concept and delineating the scope and extent of the benefits offered. As the author points out, only by establishing this definition can valid comparisons be made of different countries’ per capita expenditure on LTC. Moreover, it enables us to determine which cases should be included and which lie outside the scope of LTC. A particular aspect of LTC legislation in Spain is that dependency-related care is covered only when the lack or loss of physical, mental, intellectual or sensory autonomy which prevents the performance of basic activities of daily life is permanent. This irreversible requirement of dependency care differs from the scope of application of LTC in most other countries. One example of its impact is that it excludes certain cases when care is needed, such as the recipients of chemotherapy or radiotherapy.^3^

In defining the concept of LTC, it is essential to determine the levels of severity considered, and thus the type and intensity of dependency benefits available. For this purpose, a classification scale of dependence severity must be established, and in this respect significant disparities may arise between countries. Thus, an earlier study highlighted the contrasts between dependence-benefit scales applied in France, Germany, and Spain, showing that the Spanish scale is the most generous and the French system, the most restrictive.^4^ In view of these considerations, the homogenisation and delineation of concepts is essential for valid comparisons between countries to be made.

**Figure 1:**
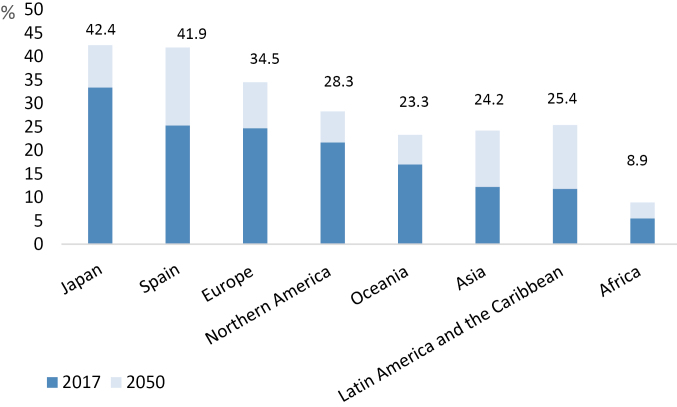
Percentage of Population Over 60 Years Old in 2017 and Projections for 2050.

However, there is an even more pressing reason to characterise the application of LTC systems, namely the exponential increase in the numbers of people who will need LTC within the coming decades. Population aging is an irrefutable fact in developed economies. Currently, Japan has the fastest-aging population in the world, with 33% of its population aged 60 years or over in 2017. By 2050, this proportion is expected to rise to 42%, closely followed by Spain with 41.9% ^5^ (see Figure 1).

Aging not only involves the loss of autonomy to perform the basic and instrumental activities of daily life, it is also characterised by the increased presence of diseases such as stroke, heart disease and dementia. Furthermore, these diseases often become chronic and multipathological.^6^ Such outcomes generate intensive financial pressures on providers of LTC, and these pressures will intensify as the proportion of the world’s population aged over 80 years rises, from 4% in 2010 to 10% in 2050.^7^ Thus, delineating and defining LTC, contextualising its present and considering its future, are questions of vital importance if benefit systems are to be adapted to address changing circumstances before they become unsustainable.

## Long-term Care in Japan and Spain and Recent Cost Containment Measures

In developed societies, the response made to changing sociodemographic patterns and the increased demand for LTC varies substantially between countries, with Japan being among the most generous systems after the Nordic countries and the United States and Spain among the least.

**Figure 2:**
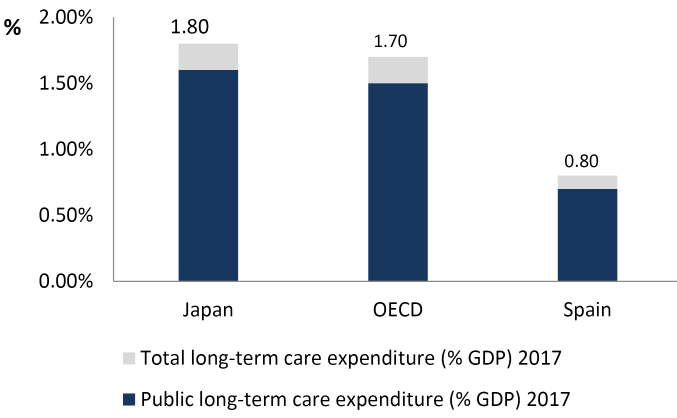
Total and Public LTC Expenditure in OECD Countries. Abbreviations: LTC, long-term care; OECD, Organisation for Economic Co-operation and Development.

As Ikegami relates in his editorial, the main approach taken to dependency care in many developed countries, including Japan, is through public insurance. The need to address LTC-related issues, outside the healthcare field, arose in Japan following the introduction of “free healthcare” in 1973 for people aged over 70 years (over 65 with disability) and the massive use of hospitals by older people. However, in many cases these hospitals functioned more as nursing homes than as healthcare institutions. Universal health coverage for LTC was introduced in 2000 under the LTC insurance (LTCI) system. In this new context, the availability of informal caregiving was not taken into consideration, in order to incentivise the use of formal services and to alleviate the care burden on the family, especially that of the daughter-in-law (traditionally the main carer of elderly relatives). In a short period of time, the provision of LTC expanded very rapidly, so much so that Japan is now one of the OECD countries with the highest LTC public expenditure per capita (see Figure 2).^1,8^ However, the financial burden associated with the sociodemographic characteristics of the Japanese population may put this system at risk in the near future.

In Spain, LTC attention has traditionally and residually been relegated to the social services, and as in Japan, prioritising people with low incomes and/or without family support. It was not until 2006 when the Spanish LTC System^9^ was formally created, following European Union recommendations to design systems of attention to the needs of the growing dependent population.^10^ The initial conception of the Spanish LTCI was to offer a list of services (nursing home, day/night centres, home help care and teleassistance) to dependents, and only in exceptional cases granting cash benefits when it was not feasible to offer a service. Cash benefits for family caregivers were originally intended to be marginal, but they became the norm rather than the exception due to the lack of formal services provided in many regions and with the stagnation of benefits following the economic recession in 2008.

Japan has made various budgetary efforts to contain spending: supplier rates and benefits have been reduced, new charges introduced and eligibility criteria tightened. However, these measures have had little impact on total LTC expenditure. LTCI co-payment was recently increased from 10% to 20% for the whole population and to 30% for people with higher incomes and assets, although this population group represents only a small proportion of the total. In Spain, efforts to contain spending and meet the objectives of fiscal consolidation were implemented in response to the severe economic recession of 2012.^11^ Among the cost containment measures introduced, monetary benefits were reduced, services were provided less intensively and the granting of benefits for low-severity dependents was delayed for three years. In parallel, measures were adopted to increase revenue collection. Thus, a new formula was employed to calculate the contribution to be made by the beneficiary, raising it to from one third of the total cost to over half.^12,13^ The new co-payment system had a sharply negative impact on household finances, which was mirrored by benefit incidence and intensity.^14^

To reduce the growing financial burden of LTC, political initiatives in Spain, Germany, and elsewhere promoted informal caregiving and the use of outpatient services to complement caregiving at home. In this respect, various caregiving allowances, including cash, pensions and workers’ compensation, were offered to encourage caregiving and alleviate its negative impact on employment and household income. However, studies have also emphasised the need for public-sector programmes and initiatives to protect and assist informal caregivers.^2,15^

The use of cash allowances is becoming an increasingly important element in the funding of LTC, worldwide, in response to rising demand and the ever-growing cost of facility-based services. However, the design of LTC benefits, and of cash allowances in particular, is a complex matter, and informal care should not be viewed as a remedy for the lack of formally-developed LTC services (as is actually the case in Spain), but rather as a question of individual or family choice. In fact, according to Europe-wide data, many citizens express a strong preference for informal care (in terms of the share of respondents stating that elderly parents are best cared for by their children, Eurobarometer survey, 2010). In addition, informal care is associated with a better quality of life,^16,17^ may substantially reduce healthcare system costs through lower rates of hospital admissions^18,19^ and safeguards households from the financial disaster that may arise when formal services are contracted.^20^

Although cultural norms in favour of informal arrangements prevail in many countries, the increasing participation of women in the labour market, together with the lack of flexible working arrangements, among other circumstances, may generate considerable obstacles to the provision of LTC within the family.^2,21^ In fact, in Germany, where the LTC system is strongly reliant on informal carers, many citizens have reported that they would rather spend less time on caregiving tasks.^22^

## Lessons and Future Challenges

As described in the previous section, the Japanese and the Spanish LTC systems were created at different stages of each country’s own history. Thus, while the Spanish LTC system was designed on an expansive economic cycle and was implemented relatively recently, the Japanese LTC system has been progressively implemented over a longer time frame and has suffered continuing reforms in these years. Moreover, the culture and customs are very different in Japan and Spain, and these aspects considerably influence the patterns of elderly care in each country. Notwithstanding the enormous complexities involved in comparing both systems of LTC, we attempt in this section to highlight some of the lessons provided by the Japanese and the Spanish LTC experience.

In the first place, LTC must be centrally coordinated with respect to a basic or minimum portfolio of benefits, and monitored to reduce discrimination. In Spain, for example, regional decentralisation has provoked significant inequalities in terms of waiting times, types of services received, and indirectly, the amounts that beneficiaries must disburse, due to the different compositions of benefits among the regions. Second, the delineation of benefits must be generous and at the same time realistic, and comply with objective and transparent criteria (as has been achieved in Japan). Third, LTC should be financed from various sources, as is the case with the Japanese LTCI system, thus reducing sensitivity to variations in the national economic cycle and enhancing the stability of the system. Finally, the financial pressures inevitably encountered in dependency systems (see Figures 1 and 2) mean that informal care and cash allowances, excessively present in the Spanish LTCI but non-existent in Japan, will often play a significant role. In fact, a key feature of the Spanish LTC system is that it covers the social contribution of informal caregivers protecting their labour rights.^23^ The optimal design of LTC benefits for countries like Japan and Spain, subject to a considerable financial burden, is a challenging and complex issue and may require them to seek a balanced combination of formal and informal care, together with broader measures to be implemented within the economy in order to promote care within the family, such as flexible working hours and other measures to reconcile family and employment obligations. As the countries currently faced by the most challenging demographic forecasts, both Japan and Spain must learn from each other’s initiatives and reforms.

## Acknowledgments

Dolores Jiménez-Rubio gratefully acknowledges funding from the Spanish Ministry of Economy and Competitiveness and the European Regional Development Fund (ECO2015-66553R).

## Ethical issues

Not applicable.

## Competing interests

Authors declare that they have no competing interests.

## Authors’ contributions

Both authors contributed equally to all aspects of this manuscript. Both authors reviewed and approved the final version.

## Authors’ affiliations

^1^Faculty of Social Sciences, University of Castilla-La Mancha, Cuenca, Spain. ^1^Department of Applied Economics, Faculty of Economics and Business Sciences, University of Granada, Granada, Spain.
